# Supplementary material for: Stakeholder Insights into Czech Performance-Based Managed Entry Agreements: Potential for Transformative Change in Pharmaceutical Access?
Source: Healthcare (Basel). 2024 Jan 4;12(1):119. doi: 10.3390/healthcare12010119 (PMC10779200; doi:10.3390/healthcare12010119)
Supplement: Supplementary file 1 [file healthcare-12-00119-s001.zip › healthcare-2766008-supplementary.pdf]

Supplementary S1: Interview Scenario

1) **Introductory Query:** Would you be willing to grant permission for the recording of this interview session?
2) **Inquiry Regarding Occupation/Position**
3) **Could you kindly specify your professional designation and delineate your responsibilities within the field?**
   - Stakeholders' Relationship with PB-MEA
   - What is the organizational role assumed by the entity you represent in the context of PB-MEA?
   - How do you personally engage with or relate to PB-MEA?
   - From your perspective, which entities or individuals are influenced by PB-MEA?
   - In your estimation, what are the merits and demerits for the involved stakeholders? Who do you believe stands to gain from PB-MEA, and who might encounter challenges?
   - Do you possess practical experience in PB-MEA agreements, or are you familiar with such instances within your milieu?
   - Do you detect any prevailing general dispositions among stakeholders towards PB-MEA, such as scepticism or enthusiasm?
4) **Legislation**
   - How do you perceive the extant legislative framework, particularly in light of the recent legislative amendment in 2022, pertaining to PB-MEA?
   - Are there discernible lacunae within the current legal framework?
   - Does there exist an impartial framework applicable to all stakeholders within the current legislative ambit?
   - Does the prevailing legislation adequately mirror societal exigencies, and is it judiciously configured?
   - In your estimation, what are the constructive and adverse facets of the current PB-MEA legal framework?
5) **Threats and Opportunities Associated with PB-MEA Implementation**
   - What opportunities do you discern within the PB-MEA domain for the stakeholders involved?
   - What threats linked to PB-MEA do you deem pertinent for the stakeholders?
   - Future Perspectives
   - What recommendations would you posit to facilitate a more efficacious PB-MEA implementation, based on your insights?
   - Which impediments necessitate resolution, and what are your foremost perceptions of the principal challenges?
6) **Outcomes**
   - How ought outcomes to be conceptually delineated, and what criteria should underpin their formulation?
   - What categories of outcomes should be deemed germane?
   - Do you evince a predilection for qualitative or quantitative parameters?
   - In what manner should outcomes be vigilantly tracked and supervised?
